# Evolutionary Genetics of Crop-Wild Complexes

**DOI:** 10.3390/genes13010001

**Published:** 2021-12-21

**Authors:** Andrés J. Cortés, Amandine Cornille, Roxana Yockteng

**Affiliations:** 1 Corporación Colombiana de Investigación Agropecuaria AGROSAVIA, C.I. La Selva, Km 7 vía Rionegro—Las Palmas, Rionegro 054048, Colombia; 2 Facultad de Ciencias Agrarias—Departamento de Ciencias Forestales, Universidad Nacional de Colombia—Sede Medellín, Medellín 050034, Colombia; 3 Université Paris-Saclay, INRAE, CNRS, AgroParisTech, GQE—Le Moulon, Gif-sur-Yvette, France; amandine.cornille@cnrs.fr or; 4 Corporación Colombiana de Investigación Agropecuaria AGROSAVIA, C.I. Tibaitatá, Km 14 vía Mosquera, Cundinamarca 250047, Colombia; ryockteng@agrosavia.co; 5 Institut de Systématique, Evolution, Biodiversité-UMR-CNRS 7205, National Museum of Natural History, 75005 Paris, France

Since Darwin’s time, the role of crop wild relatives (CWR), landraces, and cultivated genepools in shaping plant diversity and boosting food resources has been a major question [1]. After all, domestication is by far the biggest evolutionary [2] and selection trial [3] carried out by humanity. Thanks to this, we have been able to test explicit hypotheses on the patterns [4] and drivers [5] of crop domestication and diversification [6,7,8]. Despite crop wild complexes being undeniably insightful as study systems [9], and modern developments speeding up their utilization [10], studies at the crop–wild interface remain challenging. Research requires to be conducted on natural decoupling between CWR and the cultivated genepools in terms of their growing cycles, phenological phases, and dispersion strategies, not to mention the complexity derived from recurrent [11] crop–wild introgression patterns [12]. These difficulties have precluded reaching a trans-disciplinary synthesis on crop–wild systems.

Therefore, the goal of this Special Issue was to summarize fundamental and applied approaches on the evolutionary genetics in crop species and their CWR. Specifically, this compilation offers new insights into: (1) the evolutionary genetics of CWR [13] and the genomic consequences of domestication [14], (2) the role of crop–wild gene flow in adaptation [15], the utility to breed wild resources for climate change [16], and the necessity to consolidate open-source scientific networks [17] targeting underutilized/understudied plant resources.

## 1. From Early Domestication to Modern Breeding

**Figure 1:**
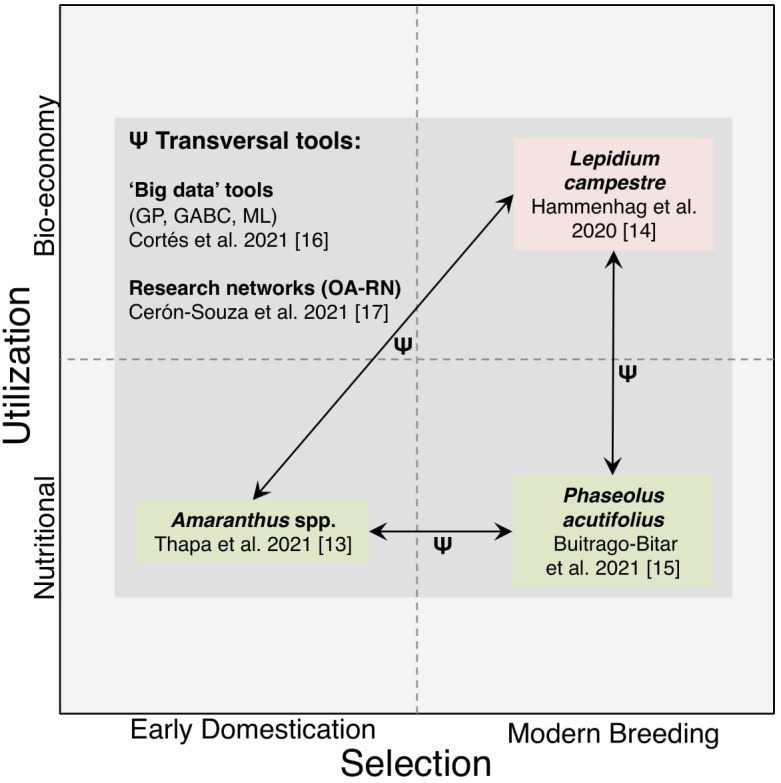
Trans-disciplinary synthesis on crop wild complexes envisioned as part of this Special Issue. Transversal ‘big data’ tools (Ψ) [16], and consolidated research networks [17], promise promoting research integration around crop wild systems across the multi-dimensional space shaped by fundamental questions (e.g., nature and timing of domestication events, *X*-axis), and more applied research (e.g., underutilized/understudied crops with potential nutritional and bio-economical value, *Y*-axis). Specifically, genomic prediction (GP) [18], genomic-assisted back-crossing (GABC), and machine learning (ML) tools [16], boosted by open-access research networks (OA-RN) [17], would allow cohesive learning from early domestication (e.g., amaranth species [13]), and modern breeding (e.g., field cress, *L. campestre* [14]). Additionally, ‘big data’ and OA-RN will enable to unlock more efficiently variation hidden at crop wild complexes with nutritional value (e.g., *Phaseolus* bean species [15]), and those with a more industrial perspective (e.g., field cress, a promising oil crop for the subarctic [14]). Based on this compilation, we encourage oncoming studies to: (1) explicitly test evolutionary scenarios concerning the domestication of the ancient grain amaranth via approximate Bayesian computation (ABC) demographic inferences [13] and ML [19,20], (2) narrow the genetic mapping of key domestication-related traits in the modern breeding of field cress [14] via genome-wide association studies (GWAS) and GP [18], and (3) utilize tepary bean as a source of adapted alleles for drought tolerance (and potentially heat tolerance) in common bean [15] via inter-specific crossing schemes [21] and more modern GABC-based indirect introgression breeding.

In an effort to unveil the domestication of an ancient grain (Figure 1), Thapa et al. [13] studied the relationship of cultivated grain amaranth species and their wild relatives across a diverse panel of 276 accessions using Kompetitive Allele Specific PCR (KASP)-based single nucleotide polymorphism (SNP) markers. The authors interpreted potential domestication events. The two Mesoamerican species *Amaranthus cruentus* and *A. hypochondriacus* were inter-crossed and distantly related to the South American species *A. caudatus* and the weedy relative *A. quitensis*, both persisted in a wild-cultivated hybrid state. Future studies must validate these scenarios using demographic inferences.

Similarly, Hammenhag et al. [14] reconstructed the genomic architecture of recent ongoing domestication in field cress (*Lepidium campestre*), a promising oil crop for the subarctic. The authors genotyped 380 genotypes of an F_2_ mapping population, and its F_3_ progenies, for a total of 2330 GBS-derived SNP markers. This dataset allowed capturing nine quantitative trait loci (QTLs) linked with key domestication-related traits such as plant height, number of stems per plant, stem growth orientation, and perenniality.

## 2. Natural Adaptation Meets Breeding for Abiotic Stress Tolerance

Buitrago-Bitar et al. [15] perceptively explored allelic variation at three families of candidate genes (i.e., ASR [22], DREB [23], and ERECTA [24]) across naturally drought-tolerant legume resources from tepary bean (*Phaseolus acutifolius*), and its CWR. The team found that wild tepary offers a reservoir of unique alleles at genes for drought tolerance, surpassing conventional common bean (*P. vulgaris*) resources [25], as predicted for a species that originated in arid climates at the Mexico–USA border [26]. This research has already served as the foundation to counterbalance the domestication winnowing effect on natural genetic variation [27] via inter-specific crossing schemes [21].

However, a question remains open: how can we more efficiently unlock naturally available diversity from CWR and landraces as part of pre-breeding efforts? [28]. With this perspective in mind, novel genomic-based [29] strategies are reviewed here [16] to better utilize natural adaptation from CWR genepools [30]. The authors argue that adaptation of CWR to hot and dry climates is indicative of how plants could respond to extreme weather [31]. Since natural selection has already tested more options than humans ever will [32], the review proposes (1) habitat-based population-guided samplings targeting unexplored semi-arid regions, and (2) geo-referencing-based environmentally coupled genetic characterizations of those collections [33,34]. The review ends prospecting last-generation ‘big data’ pipelines, such as genomic prediction [18], genomic-assisted back-crossing (GABC), speed breeding [10], and machine learning [35], all of which may help CWR boost pre-breeding for adaptation [36,37].

## 3. Meeting Future Demands

Balancing future nutritional [38] and bio-economical needs requires powering ‘big data’ [39] integrative trans-disciplinary strategies [40]. Precisely, all contributions to this Special Issue coincided with the need to merge the phylogenetic diversity [41], conservation efforts [42], and innovative utilization [43] of crop wild complexes to avoid plant resources becoming obsolete. Still, the bottlenecks promoting these synthetic approaches include the availability of open-source data [44], and consolidated research networks. In this sense, Cerón-Souza et al. [17] have called for a very timing moratorium to define guiding principles that could enhance research impact around plant genetic resources by bridging strong fragmentation and low connectivity among teams. The pillars of such discussion include the need to: (1) monitor and forecast gender and generation gaps to shrink disparity over time, (2) fund long-term synergies to leverage plant resources independent of market trends [45], (3) bridge plant germplasm resources with plant breeding [46], and (4) encourage joint training programs in last-generation technologies to speed up breeding cycles [18] and mitigate tradeoffs [47]. In parallel, it is necessary to find innovative bio-economical uses (such as was carried out by Hammenhag et al. [14]), and to (5) harness neglected or underutilized species [48] as a source of new [49] and future [37] crops (as envisioned by Buitrago-Bitar et al. [15] and Thapa et al. [13]). We look forward to seeing future research implementing these recommendations on crop–wild complexes [50,51,52,53].

## Data Availability

For original datasets, please refer to the published articles [13,14,15,16,17] within the Special Issue “Evolutionary Genetics of Plant Crop-Wild Complexes: From Fundamental to Applied Research” (https://www.mdpi.com/journal/genes/special_issues/Plant_Crop_Wild).
